# Novel inflammatory biomarkers associated with stroke severity: results from a cross-sectional stroke cohort study

**DOI:** 10.1186/s42466-023-00259-3

**Published:** 2023-07-20

**Authors:** Lino Braadt, Markus Naumann, Dennis Freuer, Timo Schmitz, Jakob Linseisen, Michael Ertl

**Affiliations:** 1 Department of Neurology and Clinical Neurophysiology, University Hospital Augsburg, Augsburg, Germany; 2 Epidemiology, Faculty of Medicine, University of Augsburg, Augsburg, Germany

**Keywords:** Stroke, Biomarkers, Functional outcome, Stroke severity

## Abstract

**Background:**

Stroke is a leading cause of mortality and disability worldwide and its occurrence is expected to increase in the future. Blood biomarkers have proven their usefulness in identification and monitoring of the disease. Stroke severity is a major factor for estimation of prognosis and risk of recurrent events, but knowledge on respective blood biomarkers is still scarce. Stroke pathophysiology comprises a multitude of ischemia-induced inflammatory and immune mediated responses. Therefore, the assessment of an immune-related panel in correlation with stroke severity seems promising.

**Methods:**

In the present cross-sectional evaluation, a set of 92 blood biomarkers of a standardized immune panel were gathered (median 4.6 days after admission) and related to stroke severity measures, assessed at hospital admission of acute stroke patients. Multivariable logistic regression models were used to determine associations between biomarkers and modified Rankin Scale (mRS), linear regression models were used for associations with National Institute of Health Stroke Scale.

**Results:**

415 patients (mean age 69 years; 41% female) were included for biomarker analysis. C-type lectin domain family 4 member G (CLEC4G; OR = 2.89, 95% CI [1.49; 5.59], *p*_*adj*_ = 0.026, Cytoskeleton-associated protein 4 (CKAP4; OR = 2.38, 95% CI [1.43; 3.98], *p*_*adj*_ = 0.019), and Interleukin-6 (IL-6) (IL6; OR = 1.97, 95% CI [1.49; 2.62], *p*_*adj*_ < 0.001) were positively associated with stroke severity measured by mRS, while Lymphocyte antigen 75 (LY75; OR = 0.37, 95% CI [0.19; 0.73], *p*_*adj*_ = 0.049) and Integrin alpha-11 (ITGA11 OR = 0.24, 95% CI [0.14, 0.40] *p*_*adj*_ < 0.001) were inversely associated. When investigating the relationships with the NIHSS, IL-6 (β = 0.23, 95% CI [0.12, 0.33] *p*_*adj*_ = 0.001) and ITGA11 (β =  − 0.60, 95% CI [− 0.83, − 0.37] *p*_*adj*_ < 0.001) were significantly associated.

**Conclusions:**

Higher relative concentrations of plasma CLEC4G, CKAP4, and IL-6 were associated with higher stroke severity, whereas LY75 and ITGA11 showed an inverse association. Future research might show a possible use as therapeutic targets and application in individual risk assessments.

**Supplementary Information:**

The online version contains supplementary material available at 10.1186/s42466-023-00259-3.

## Introduction

Stroke is a leading cause of mortality and disability worldwide. Despite recent advancements in acute stroke management, such as thrombolysis and mechanical thrombectomy, the risk of early neurological deterioration remains a substantial risk [1] and long-term outcomes for stroke survivors remain poor. Additionally, stroke recurrence rates have not changed over the past 20 years [2].

While scores, such as the ABCD2-score, aim at risk stratification for recurrent events, there is evidence [3] that such few factors do not depict the biological complexity behind the risk of cerebral ischaemia and are therefore pose significant limitations. Outcome prediction is an important part of stroke care in the acute, subacute and chronic phase. It enables planning of rehabilitation goals and offers individualized and also resource-efficient programmes [4].

In the future, artificial intelligence and machine learning-based models are likely to assist physicians estimating the individual patient prognosis. Algorithms, such as the deep neural network, have proven to be effective e.g., in prediction of long-term neurological outcomes [5] or early neurological deterioration [6]. The quality of this technology depends on the number and the specificity of provided variables [7]. Blood biomarkers specifically associated with stroke severity might play an important role in these circumstances.

Several blood biomarkers have been investigated in stroke research, including markers of inflammation, coagulation, oxidative stress, and neuronal injury. Inflammation plays a crucial role in stroke pathophysiology, and several studies have investigated the association between inflammatory biomarkers and stroke outcomes. Elevated levels of inflammatory biomarkers, such as C-reactive protein (CRP), interleukin-6 (IL-6), and tumor necrosis factor-alpha (TNF-α), have been associated with poor stroke outcomes, including increased mortality, disability, and recurrent stroke risk [8–10].

Despite the growing body of evidence on blood biomarkers in stroke, the majority of studies have investigated only a limited number of biomarkers and their association with stroke severity and outcomes. Therefore, there is a need for comprehensive biomarker profiling to identify novel biomarkers and elucidate their role in stroke pathophysiology. Furthermore, by expanding the spectrum of significant biomarkers associated with stroke severity, outcome and risk of recurrence, machine learning methods might gain higher precision in their prediction abilities. As inflammation plays a major role in stroke pathophysiology, we investigated a panel of immune-related blood biomarkers and their association with stroke severity and outcomes in this study.

## Material and methods

### Study population and data collection

**Fig. 1:**
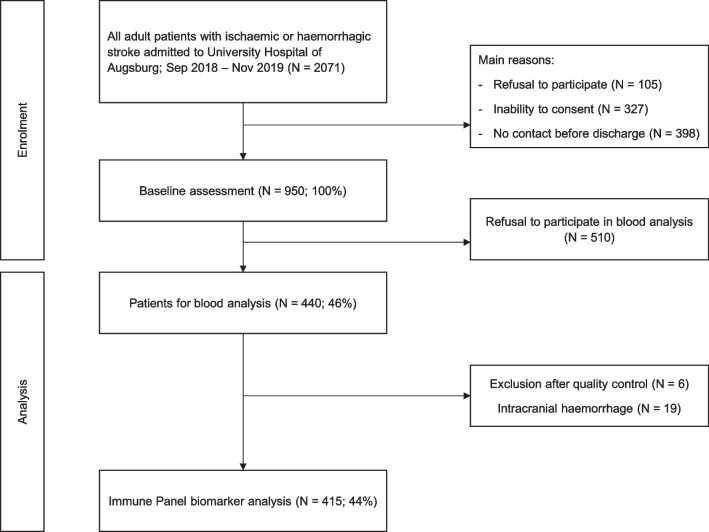
Consort chart of patient enrolment and analysis

All adult patients admitted with ischemic or hemorrhagic strokes or transient ischemic attacks (TIA) to the University Hospital of Augsburg between September 2018 and November 2019 were screened for inclusion in this stroke cohort. Details of enrolment, methods, conduction of interviews as well as follow-up data have been published elsewhere [11]. In summary, study nurses recorded all stroke cases and excluded those, who refused to consent or were unable to do so. A share of patients was missed before discharge, e.g. because of premature discharge. After having received written informed consent, 44% of all patients were included and baseline interviews and chart reviews were performed. Hereby information about general biography (including age and sex), diagnoses, laboratory findings, treatment, comorbidities and education were gathered as possible confounders. The educational level was dichotomized into “low” and “high” by using International Standard Classification of Education (ISCED)-scores 1–3 and 4–6. This method had already been used elsewhere [12, 13]. Blood was taken, from those patients who gave their consent, during hospital stay and stored serum aliquots were used for the analysis of the “Olink Target 96 Immune Response” panel [14] (see consort chart, Fig. 1). In our analysis we focused on ischemic strokes because of different mechanisms in pathophysiology in comparison to intracerebral bleedings.

### Outcome evaluation

Stroke severity was measured using the National Institutes of Health Stroke Scale (NIHSS; ranging from 0 to 42) [15] and the modified Rankin Scale (mRS; ranging from 0 to 6) [16], both were recorded upon hospital admission and discharge. For our present analysis, we focused on the stroke severity at admission, possible acute treatments and individual recovery. The mRS-measures were dichotomized into mRS 0–2 = group 1, 3–5 = group 2. For assessment of protein biomarkers, we used the “Olink Target 96 Immune Response” panel: analyses were carried out by Proximity Extension Assays (PEA) with quantitative polymerase chain reaction (qPCR) readouts [17].

We included only those biomarkers, which were present in at least 25% of patients. Thereby we moved forward to statistical analysis with 63 out of 92 biomarkers.

Inflammatory parameters at admission were measured with C-reactive protein (CRP), measured in mg/dL and leukocyte counts per nL. CRP concentrations of ≥ 0.5 mg/dL and leucocyte counts > 10 leukocytes/nL were regarded as increased.

### Statistical analysis

Continuous variables were described by median and the respective interquartile range (IQR), while categorical variables were presented as absolute and relative frequencies. Differences between both mRS groups were assessed using the Mann–Whitney-U test and Pearson’s $\chi^{2}$-test for continuous and categorical variables, respectively.

To investigate the associations between biomarkers and mRS as proxy for stroke severity we performed multivariable logistic regression models. Associations with NIHSS as severity were investigated performing linear regression models. Due to the assumption of normally distributed residuals, a square root transformation was applied to the NIHSS. Heteroscedasticity was assessed by visual evaluation of the residual plots and by the Breusch-Pagan test.

Possible confounders were selected using a directed acyclic graph (DAG) together with the disjunctive cause criterion. Therefore, all models were adjusted for age, sex, BMI, stroke etiology (macroangiopathic, cardiogenic, microangiopathic, cryptogenic and other etiologies), smoking status (current, ex, never), education level (low, high), sobriety (yes, no), recurrent stroke (yes, no), hypertension (yes, no), alteplase treatment (yes, no), and time between admission and blood collection. To minimize residual confounding, continuous variables were treated as such and the linearity assumption was tested and, if necessary, adapted applying restricted cubic splines. Briefly, in each individual model the variable-specific number of knots between 3 and 5 were compared with regard to Akaike’s and Bayesian information criterions (AIC, BIC) and tested against the respective basic model (in which the respective variable were included in a linear way) using the Likelihood ratio test. Finally, we calculated the variance inflation factor (VIF) and assessed the Durbin-Watson statistic to ensure that there was no multicollinearity or autocorrelation.

To exclude patients with relevant clinical infections, possibly due to complications such as pneumonia or urinary tract infection, sensitivity analyses for all associations were performed considering only participants with a CRP $\le$ 3 mg/dl.

With regard to multiple testing, *p* values from regression models were adjusted for the false discovery rate (FDR). All mentioned above analyses were done using the statistical Software R (version 4.2.1).

Correlations between inflammatory parameters (CRP and leukocytes) and biomarkers were investigated using Spearman-correlation. Non-normal distribution was confirmed using QQ-plots and Shapiro–Wilk tests. The latter analyses were done using the statistical software SPSS (version 28.0.0.0). Statistical tests were performed two-sided at the significance threshold $\alpha = 0.05$.

## Results

### Baseline characteristics

We included 950 consecutive patients of whom 440 gave their consent for blood sample collection and analysis. From these, six patients were excluded after sample quality control and 19 patients were excluded due to intracranial hemorrhage. A total of 415 samples were sent to the external laboratory of Olink. Baseline characteristics are summarized in Table 1. A total of 412 patients had documented mRS and these were differentiated into two groups of severity, as mentioned above (group 1 n = 221 vs. group 2 n = 190). Age (median 68 (58; 78) vs. 75 (65.5; 80), *p* =  < 0.001), sex (*p* = 0.014), depression (*p* = 0.019) and stroke etiology (*p* =  < 0.001) differed significantly between the two mRS groups. Furthermore, the days past after admission and blood sample withdrawal differed significantly among both mRS-groups (4.188 (3.08–5.402) vs. 4.888 (3.495–6.593), *p* < 0.001), see Table 2. Regarding specific etiologies, the highest severity was observed in patients with cardiogenic strokes (60 (0.321)) and the least functional impairment was caused by strokes of “cryptogenic and other” stroke etiology (86 (0.415)).

**Table 1:** Baseline characteristics of acute stroke patients, given as median (IQR) or absolute and relative frequency

Characteristics	Number of patients	Median (IQR) or n (%)
Age (years)	415	71 (60; 79)
BMI (kg/m^2^)	408	26.56 (23.88; 30.06)
NIHSS	411	1 (0; 4)
Fasting status	413	
No		383 (0.927)
Yes		30 (0.073)
Time (days) between admission and blood collection	412	4.625 (3.3; 5.85)
mRS at admission	411	
Group 1 (0–2)		221 (0.538)
Group 2 (3–5)		190 (0.462)
mRS at discharge	410	
Group 1 (0–2)		332 (0.81)
Group 2 (3–5)		78 (0.19)
Sex	415	
Male		243 (0.586)
Female		172 (0.414)
Smoking status	415	
Current		75 (0.181)
Ex-smoker		170 (0.41)
Never		170 (0.41)
Depression	394	
No		360 (0.914)
Yes		34 (0.086)
Educational level	394	
Low		304 (0.772)
High		90 (0.228)
Aetiology	397	
Macroangiopathy		90 (0.227)
Cardiogenic		98 (0.247)
Microangiopathy		76 (0.191)
Cryptogenic and other		133 (0.335)
Recurrent stroke	414	
No		308 (0.744)
Yes		106 (0.256)
Arterial hypertension	415	
No		83 (0.2)
Yes		332 (0.8)
CRP (mg/dL)	406	0.23 (0.11; 0.62)
Leukocytes (/nL)	415	7.81 (6.63; 9.65)

*BMI* Body mass index, *NIHSS* National Institutes of Health Stroke Scale, *mRS* Modified Rankin Scale, *tmdelta* Days between admission and gathering of sample

**Table 2:** Comparison patient characteristics by mRS at admission, given as median (IQR) or absolute and relative frequency

Characteristics	n total = 411	mRS 0–2 n = 221	mRS 3–5 n = 190	*p* value
Age	411	68 (58; 78)	75 (65; 80)	** < 0.001**
BMI	404	26.83 (23.84; 29.73)	26.37 (24.2; 30.54)	0.627
NIHSS	411	1 (0; 1)	4 (2; 8)	** < 0.001**
Days between admission and blood taking	408	4.188 (3.08; 5.402)	4.888 (3.495; 6.593)	** < 0.001**
Fasting status	409			0.743
Fasting at admission		203 (0.923)	176 (0.931)	
Not fasting at admission		17 (0.077)	13 (0.069)	
Sex	411			**0.017**
Male		142 (0.643)	100 (0.524)	
Female		79 (0.357)	90 (0.474)	
Smoker	411			0.278
Current		46 (0.208)	28 (0.147)	
Ex-smoker		88 (0.398)	82 (0.432)	
Never		87 (0.394)	80 (0.421)	
Depression	391			**0.019**
No		201 (0.944)	156 (0.876)	
Yes		12 (0.056)	22 (0.124)	
Educational level	391			0.274
Low		160 (0.751)	142 (0.798)	
High		53 (0.249)	36 (0.202)	
Aetiology	393			** < 0.001**
Macroangiopathy		45 (0.217)	44 (0.237)	
Cardiogenic		36 (0.174)	60 (0.323)	
Microangiopathy		40 (0.193)	36 (0.194)	
Cryptogenic and other		86 (0.415)	46 (0.247)	
Recurrent stroke	410			0.595
No		166 (0.755)	139 (0.732)	
Yes		54 (0.245)	51 (0.268)	
Arterial hypertension	411			0.176
No		49 (0.222)	32 (0.168)	
Yes		172 (0.778)	158 (0.832)	

Analysis of continuous variables: Mann–Whitney-U test, Analysis of categorical variables: Pearson’s Chi-squared test. *p*-values of < 0.05 are written in bold

*BMI* Body mass index, *NIHSS* National Institutes of Health Stroke Scale, *mRS* Modified Rankin Scale, *tmdelta* Days between admission and gathering of sample

### Proteins of the immune panel

**Fig. 2:**
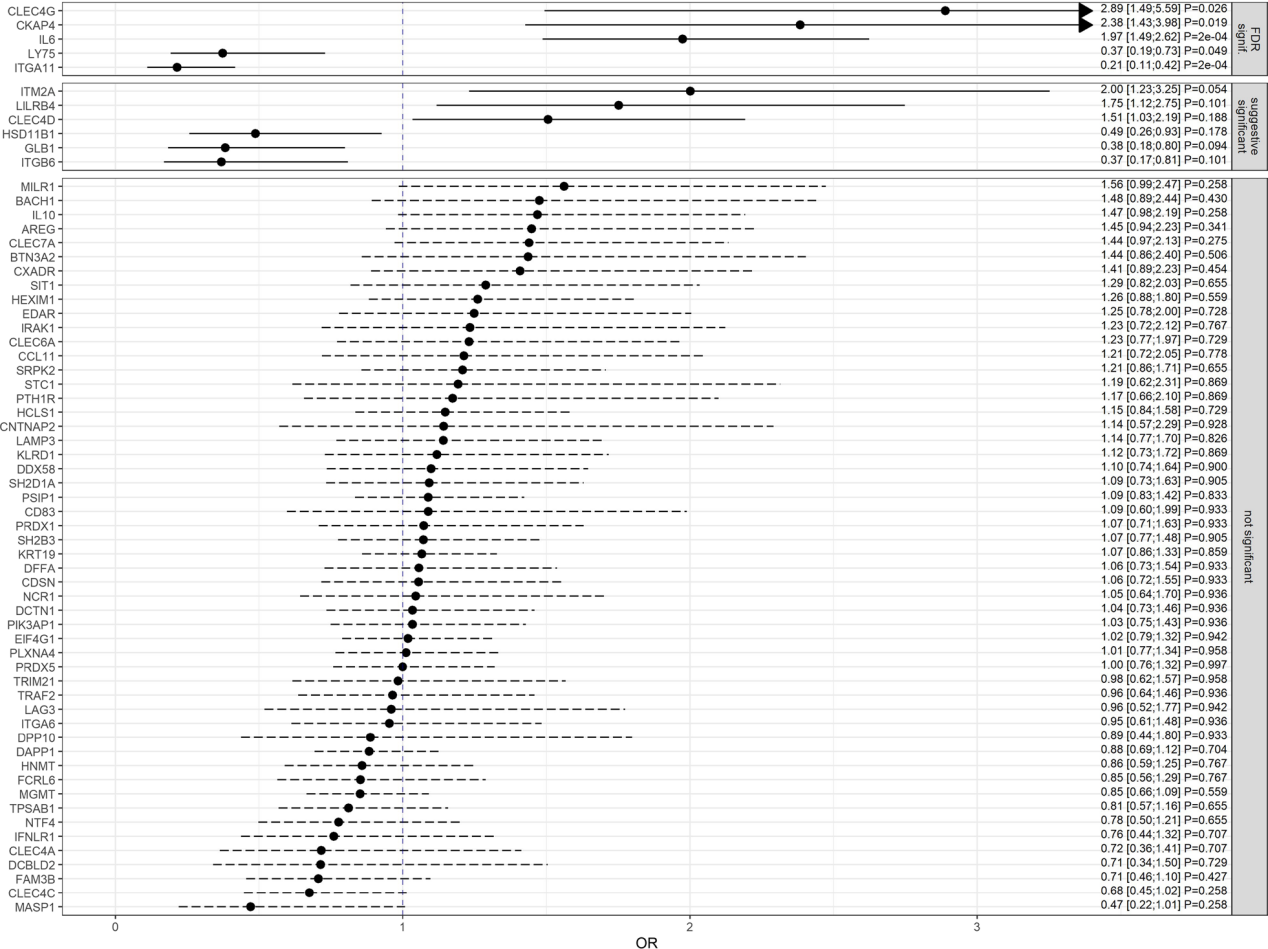
Odds ratios and 95% confidence intervals for the association between immune biomarkers and mRS (0–2 versus > 2). Presented *p* values are FDR-adjusted. Arrows represent confidence intervals exceeding the plotted x-range

**Fig. 3:**
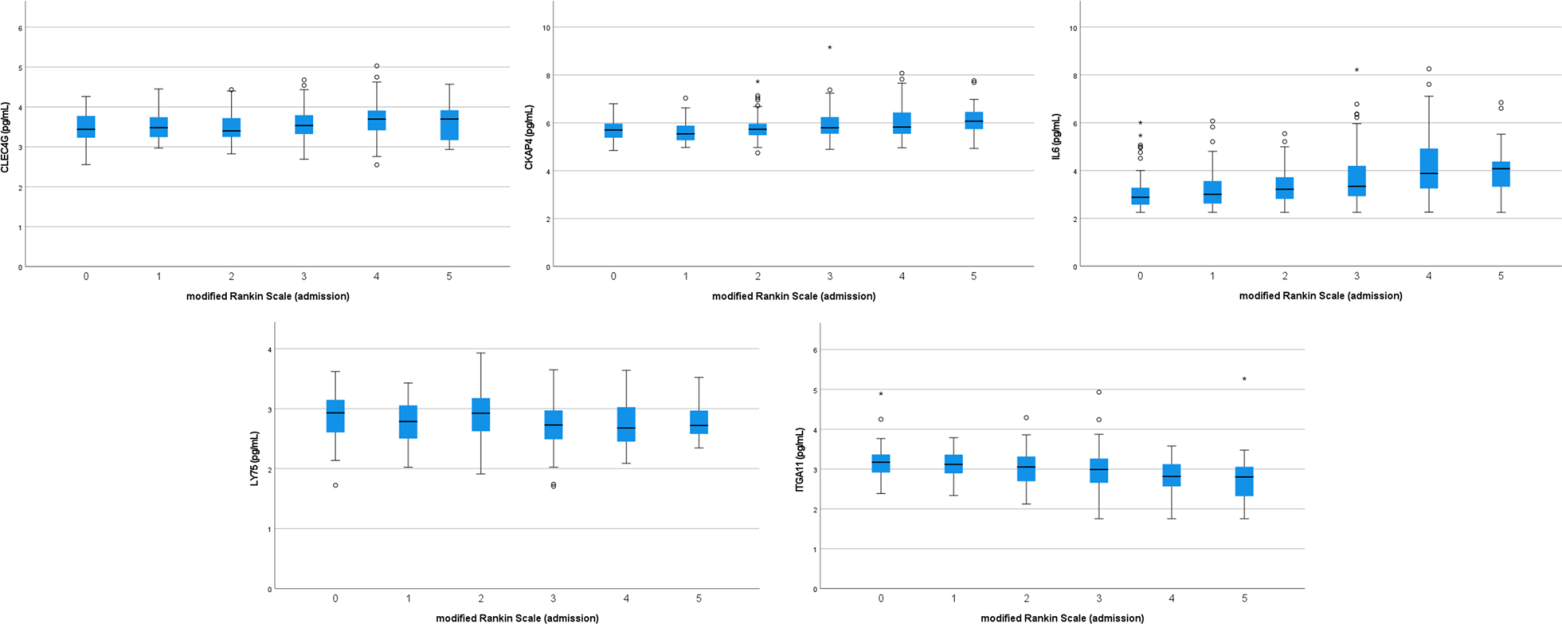
Relative plasma biomarker concentrations by mRS group (* outliers +/− 3 * IQR)

**Fig. 4:**
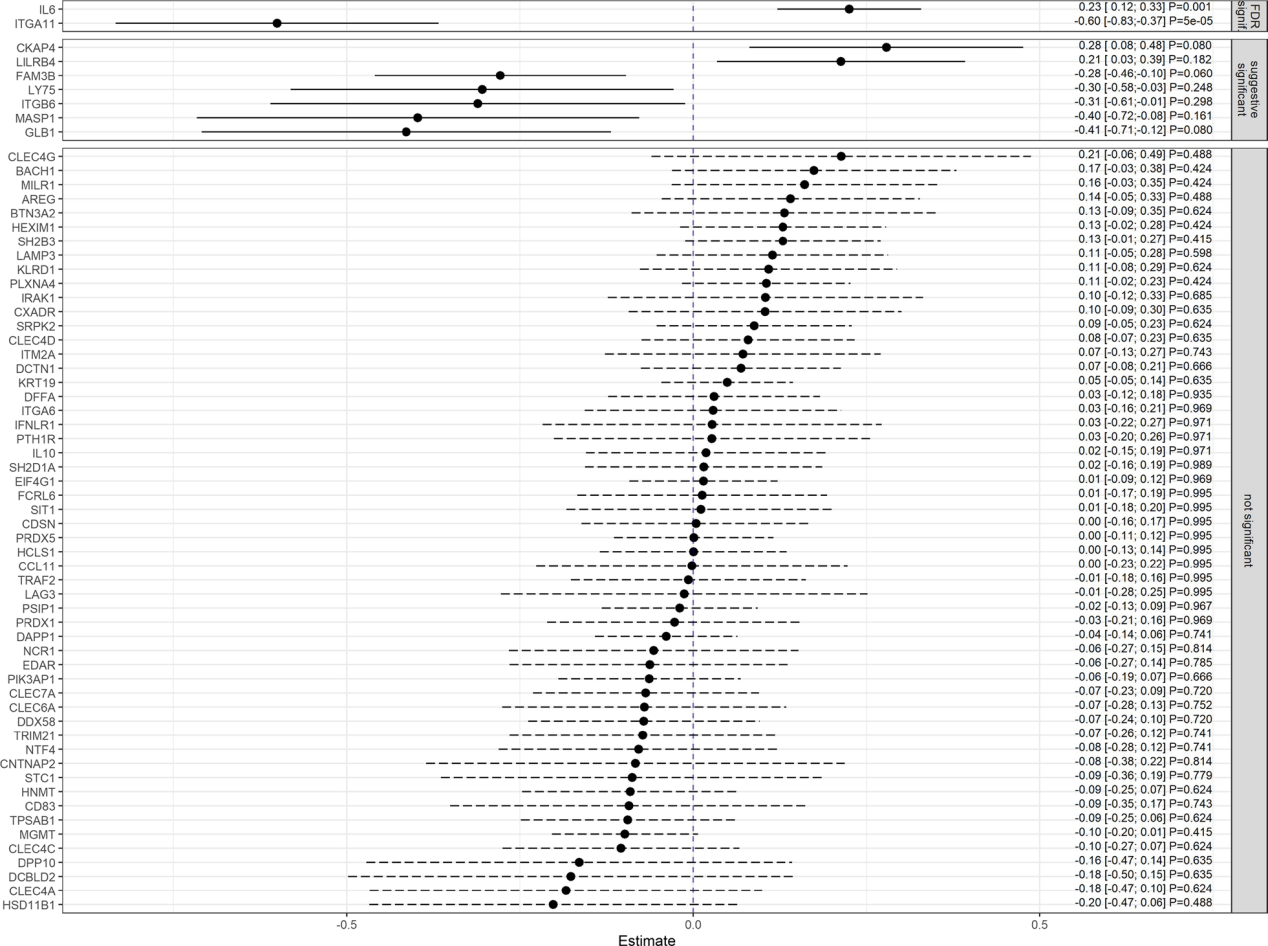
β estimates and 95% confidence intervals for the association between immune biomarkers and the square root transformed NIHSS. Presented *p* values are FDR-adjusted

For associations between biomarkers and stroke severity, we analyzed mRS and NIHSS separately. After adjustment, results were rated as “not significant”, “suggestive significant” (i.e. losing statistical significance after correction) and “significant” after correction for multiple testing by using the FDR approach. The latter was found between mRS and the biomarkers C-type lectin domain family 4 member G (CLEC4G; OR = 2.89, 95% CI [1.49; 5.59], *p*_*adj*_ = 0.026, Cytoskeleton-associated protein 4 (CKAP4; OR = 2.38, 95% CI [1.43; 3.98], *p*_*adj*_ = 0.019),, Interleukin-6 (IL6; OR = 1.97, 95% CI [1.49; 2.62], *p*_*adj*_ < 0.001), Lymphocyte antigen 75 (LY75; OR = 0.37, 95% CI [0.19; 0.73], *p*_*adj*_ = 0.049) and Integrin alpha-11 (ITGA11; OR = 0.21, 95% CI [0.11; 0.42], *p*_*adj*_ < 0.001) (see Figs. 2, 3). Regarding the NIHSS, the biomarkers IL6 (β = 0.23, 95% CI [0.12, 0.33] *p*_*adj*_ = 0.001) and ITGA11 (β = -0.60, 95% CI [-0.83, -0.37] *p*_*adj*_ < 0.001) were significantly associated on the square root scale as well (see Fig. 4). All results were supported by the sensitivity analyses performed in patients with a CRP $\le$ 3 mg/dl (see Additional files 1, 2). When correlating all biomarkers with each other, the majority of these showed significant correlations (see Additional file 3).

### Correlation with CRP concentration and leukocyte counts

CRP-levels at admission were positively correlated with leukocyte-levels (0.023 mg/dL (0.11–0.62) vs. 7.8/nL (6.63–9.64), *p* < 0.001, *r* = 0.277). Biomarkers, including those which were found to be significantly associated with stroke severity, were also examined for correlation with inflammatory parameters. Here, positive correlations were found for CLEC4G with CRP (*r* = 0.227, *p* =  < 0.001) and leukocytes (*r* = 0.147, *p* = 0.003), IL-6 with CRP (*r* = 0.375, *p* < 0.001) and leukocytes (*r* = 0.128, *p* = 0.009), and also with CKAP4 and CRP (*r* = 0.24, *p* =  < 0.001). Negative correlations were found between ITGA11 and CRP (*r* = − 0.308, *p* < 0.001) and leukocytes (*r* = − 0.268, *p* < 0.001). LY75 did not correlate significantly with inflammatory parameters (CRP *p* = 0.183, leukocytes *p* = 0.518). For the remainder of all biomarkers and their correlation with CRP- and leukocyte-levels see Additional file 3.

Furthermore, a significant positive correlation was found between CRP and the mRS (*r* = 0.173, *p* < 0.001), as well as the NIHSS (*r* = 0.142, *p* = 0.004). Leukocytes did not correlate significantly with a functional stroke scale.

## Discussion

The findings of this study confirm the association of certain blood biomarkers with stroke severity and also show new associations. The biomarkers CLEC4G, CKAP4, IL6, LY75 and ITGA11 were found to be significantly associated with stroke severity as measured by mRS and in case of IL6 and ITGA11 also by NIHSS. These results suggest that the levels of these biomarkers may be useful in predicting stroke severity and thus the functional outcome of stroke patients.

Prediction of stroke outcomes requires a high amount of precision, which can be gained by a combination of clinical judgement, validated scales, neuroimaging and laboratory findings. The number of specific blood biomarkers is still low but these markers might play an important role in prognostic scales or even machine-based algorithms, which have already proven to outperform conventional scores in stroke and cardiovascular risk assessment [18–21]. Regarding laboratory findings, reliable and stroke related biomarkers are of special interest in outcome prediction. Among the biomarkers in the present study, we confirmed two positive associations with stroke severity:

First, IL6, a proinflammatory cytokine, was found to be positively associated with both stroke severity as measured by NIHSS and mRS, the latter being a novel finding. One study found that IL6 levels were significantly elevated in the CSF of patients with acute ischemic stroke and were positively correlated with infarct volume and severity [22]. Another study found that IL6 levels were significantly elevated in the serum of patients with acute ischemic stroke and were positively correlated with NIHSS scores [23]. Also, lower IL6 levels at admission were have been associated with complete or near-complete reperfusion after single thrombectomy (first-pass effect) [24]. The authors suggested that IL6 may be a potential biomarker for stroke severity and may serve as a therapeutic target for reducing inflammation and tissue damage following stroke.

The second confirmed association with stroke severity is with CKAP4, although the earlier known associated score was also the NIHSS [25], the mRS represents again a new finding.

CKAP4, a cytoskeleton-associated protein, has been suggested to play a role in the regulation of the immune response and inflammation. One study found that CKAP4 expression was increased in the infarcted area of rat brains after stroke, suggesting that CKAP4 may be involved in the inflammatory response and neuronal damage following stroke [26]. Another study found that CKAP4 was upregulated in peripheral blood mononuclear cells (PBMCs) of patients with acute ischemic stroke [25]. The authors hypothesized that CKAP4 may be involved in the immune response following stroke and may serve as a potential therapeutic target.

To our best knowledge, the following biomarkers have not been correlated with cerebral damage in humans, including ischemia, before. One of these showed also a positive association with stroke severity.

CLEC4G, alternatively known as liver and lymph node sinusoidal endothelial cell C-type lectin (LSECtin), was associated with stroke severity when measured by mRS. It was found to be a regulator of T-cells, acting in hepatic T-cell immune suppression [27]. Furthermore, it is known for acting as a receptor in several viral infections, such as SARS-CoV [28], Japanese encephalitis virus [29], Lassa virus [30] and lymphocytic choriomeningitis virus [31]. All viruses have in common, that they can cause infection of the central nervous system and thus possibly causing structural damage of the brain.

The remaining two biomarkers showed negative associations with stroke severity, indicating their possible protective role in stroke pathophysiology:

LY75, also known as DEC-205, was negatively associated with the mRS. It is an antigen-uptake receptor on dendritic cells [32]. In other studies, LY75 was found to participate in controlling cellular phenotypes in breast cancer and thus their metastatic potential [33]. Regarding environmental factors, DEC-205, together with IL-6 and CD86, was found to be predictive biomarker for respiratory and immune effects of particulate matter [34].

At last, ITGA11 showed a negative association with stroke severity as measured by mRS and NIHSS. ITGA11 belongs to a family of collagen receptors, which have been shown to play a role in fibrosis and tissue repair. A similar integrin molecule (ITGA4) was found to be upregulated in the peri-infarct area of rat brains after stroke and that its inhibition can reduce ischemic brain injury [35]. Therefore, ITGA11 may indeed have a protective role in stroke pathophysiology and may serve as a potential therapeutic target for promoting tissue repair and recovery following stroke.

### Strengths and limitations

The analysis of a complete immune panel of biomarkers in a large cross-sectional stroke cohort is a major strength of the present study. Despite the “immune-specificity” of the panel, there are many gaps in knowledge about associations between these biomarkers and central nervous processes, especially stroke.

As limitations, from only 415 out of 950 patients (44%) blood samples were obtained. Many patients who could not be included in the study suffered from potentially more severe strokes (e.g. inability to consent, refusal by caregivers). The time gap between analysis of routine inflammatory parameters (CRP, Leukocytes) and biomarkers is a limitation of the analysis of their correlation. Additionally, the time point for blood sampling for biomarker analysis was not standardized and the delay between the stroke and blood sampling, due to factors such as delayed consent by caregivers, constitutes another limitation of the present study. With a cross-sectional study design, we could not proof a cause-effect-relationship.

## Conclusion

The present study confirmed the positive associations between stroke severity and IL6 and CKAP4, but also elucidated novel associations, namely between CLEC4G, LY75 and ITGA11, which enrich the present knowledge of blood biomarkers associated with stroke.

LY75 and ITGA11 were associated with lower stroke severity and might serve as potential therapeutic targets for promoting tissue repair and recovery following stroke.

## Supplementary Information

**Additional file 1: Figure S1.** Odds ratios and 95% confidence intervals for the association between immune biomarkers and mRS. Presented *p* values are FDR-adjusted. The black-colored estimates represent the results of the main analyses, and the gray-colored ones those of the sensitivity analyses i.e., after exclusion of patients with serum CRP > 3 mg/dl. The left panel shows the associations with a *p* value < 0.05 and the right panel shows the associations with a *p* value ≥ 0.05.

**Additional file 2: Figure S2.** β estimates and 95% confidence intervals for the association between immune biomarkers and the square root transformed NIHSS. Presented *p* values are FDR-adjusted. The black-colored estimates represent the results of the main analyses, and the gray-colored ones those of the sensitivity analyses i.e., after exclusion of patients with serum CRP > 3 mg/dl. The left panel shows the associations with a *p* value < 0.05 and the right panel shows the associations with a *p* value ≥ 0.05.

**Additional file 3: Table S1.** Correlations between all immune protein biomarkers, C-reactive protein and leukocytes.

## Data Availability

The datasets used and analysed during the current study are available from the corresponding author on reasonable request.
