# Supplementary figures and images for: Novel inflammatory biomarkers associated with stroke severity: results from a cross-sectional stroke cohort study
Source: Neurol Res Pract. 2023 Jul 20;5:31. doi: 10.1186/s42466-023-00259-3 (PMC10357843; doi:10.1186/s42466-023-00259-3)

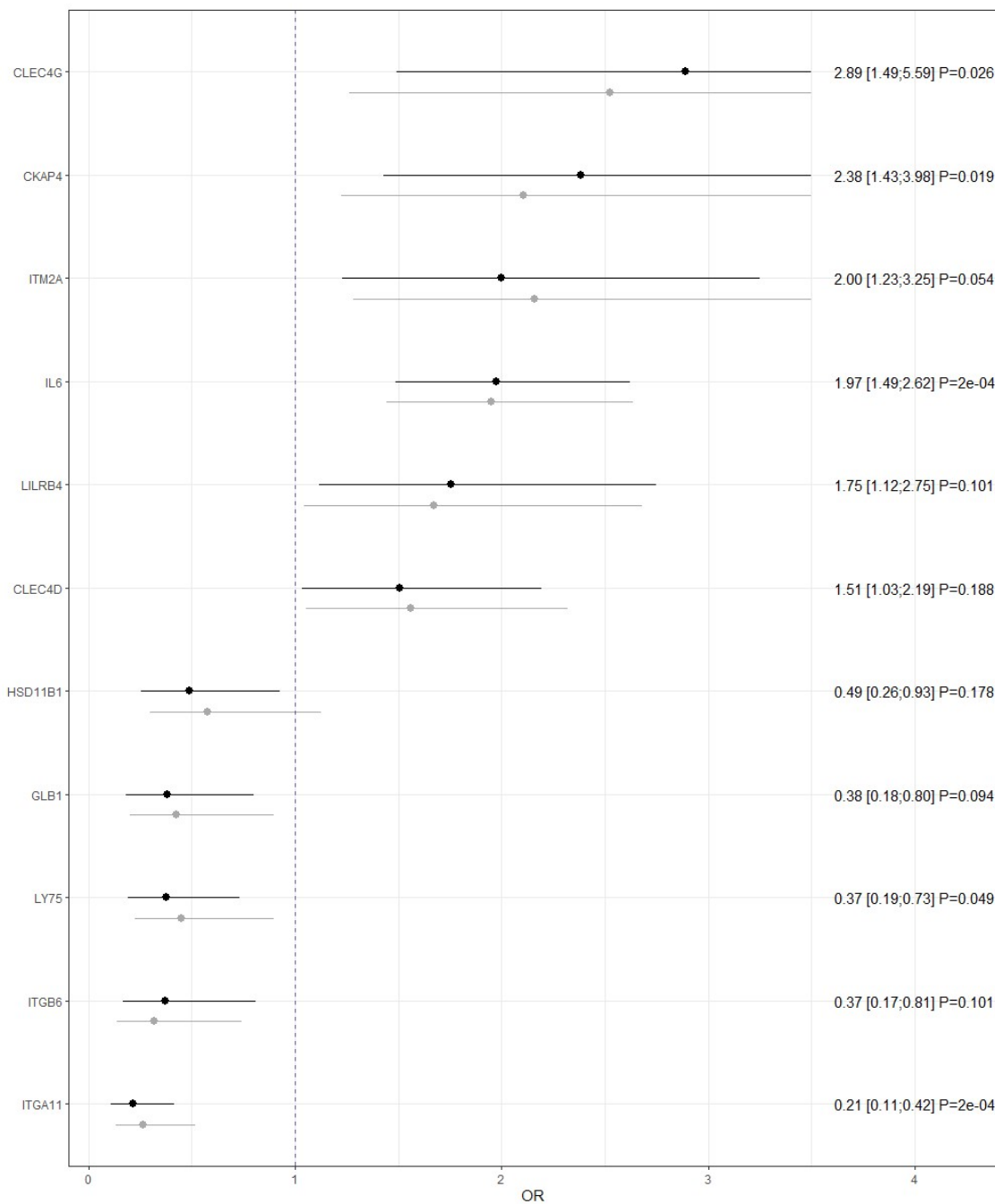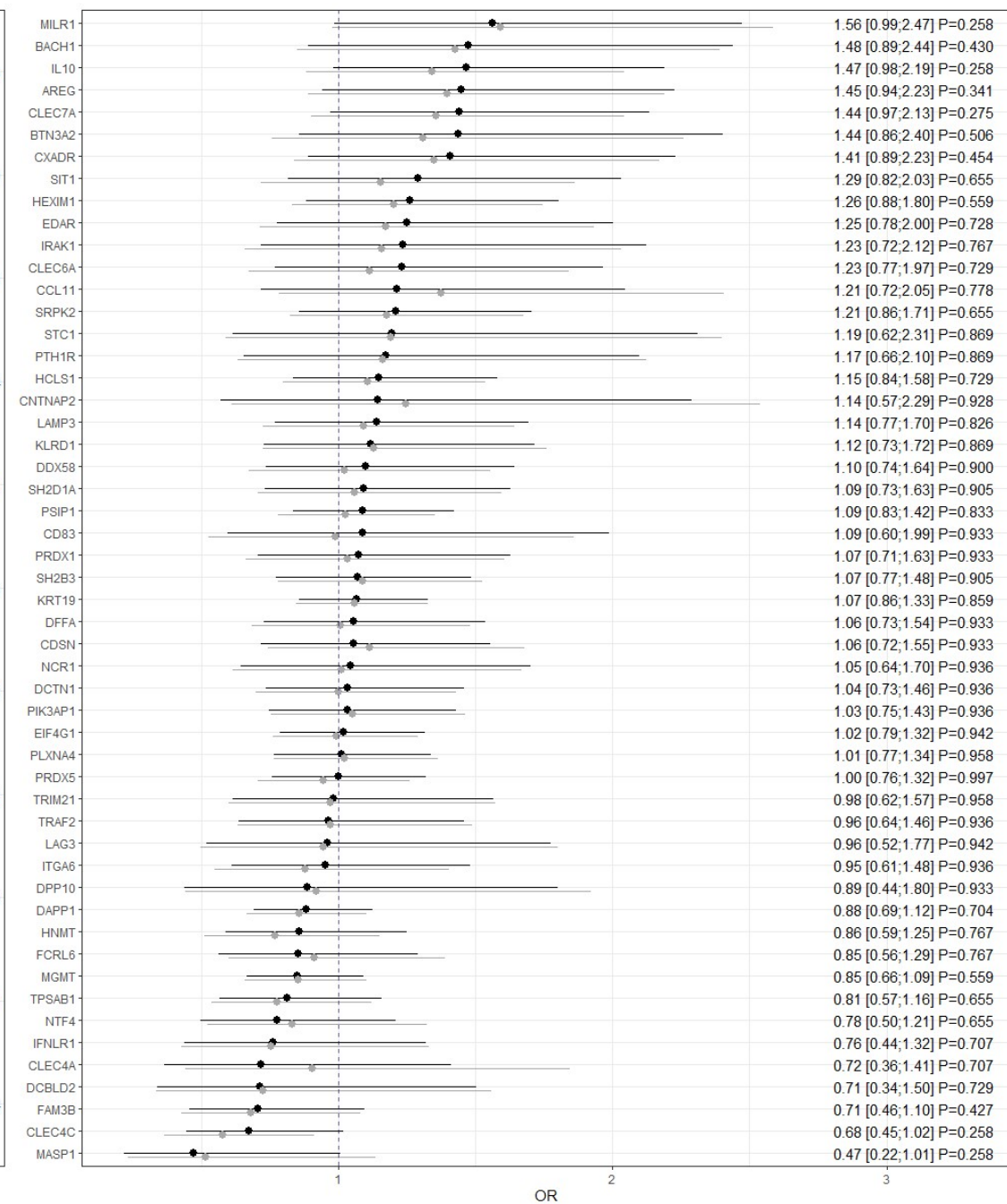

Supplement: Supplementary file 1 — Additional file 1: Figure S1. Odds ratios and 95% confidence intervals for the association between immune biomarkers and mRS. Presented p values are FDR-adjusted. The black-colored estimates represent the results of the main analyses, and the gray-colored ones those of the sensitivity analyses i.e., after exclusion of patients with serum CRP > 3 mg/dl. The left panel shows the associations with a p value < 0.05 and the right panel shows the associations with a p value ≥ 0.05. [file 42466_2023_259_MOESM1_ESM.pdf]

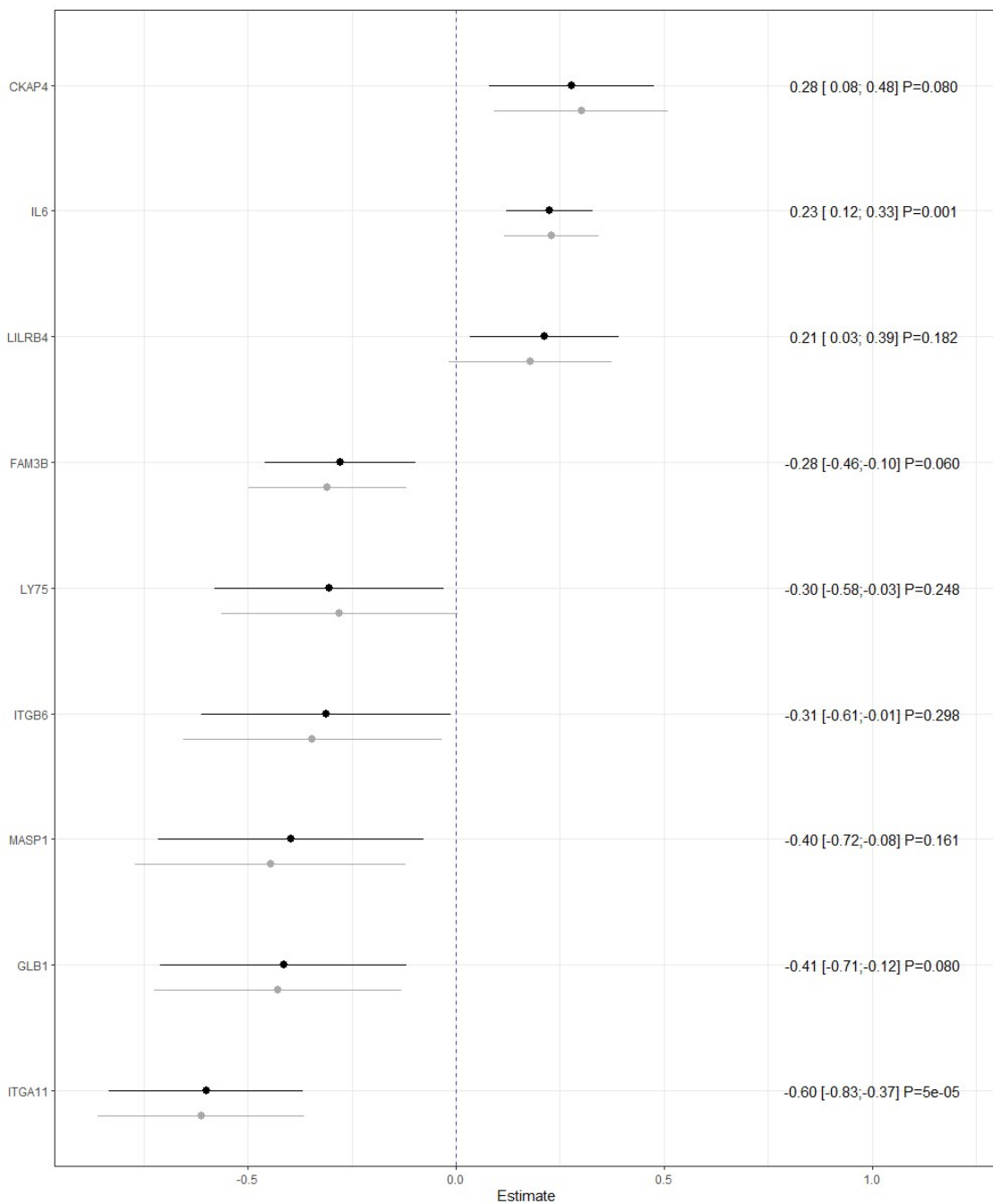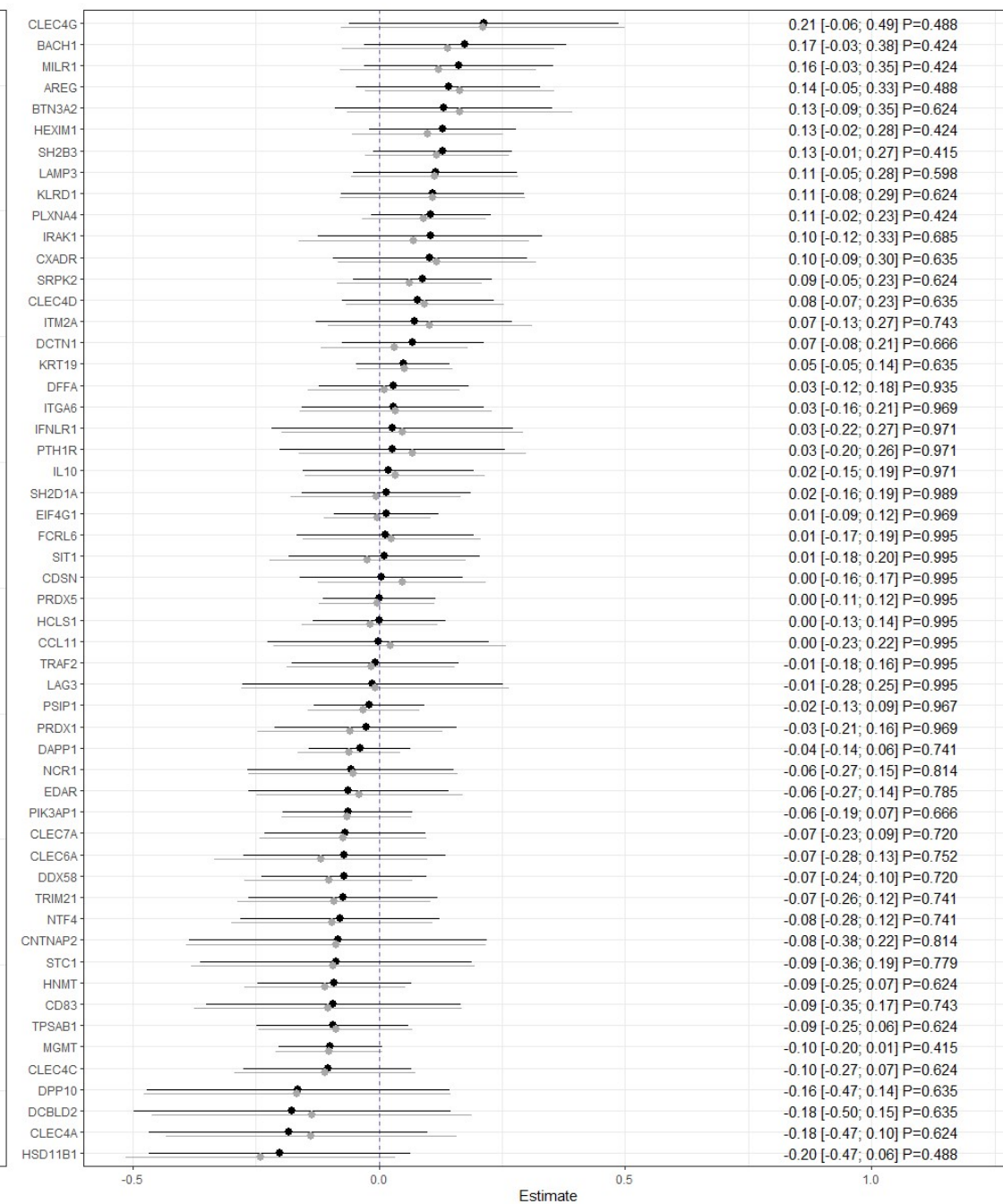

Supplement: Supplementary file 2 — Additional file 2: Figure S2. β estimates and 95% confidence intervals for the association between immune biomarkers and the square root transformed NIHSS. Presented p values are FDR-adjusted. The black-colored estimates represent the results of the main analyses, and the gray-colored ones those of the sensitivity analyses i.e., after exclusion of patients with serum CRP > 3 mg/dl. The left panel shows the associations with a p value < 0.05 and the right panel shows the associations with a p value ≥ 0.05. [file 42466_2023_259_MOESM2_ESM.pdf]
